# Preoperative Soluble AXL in Plasma Predicts Futility of Resecting Pancreatic Ductal Adenocarcinoma

**DOI:** 10.3390/curroncol33020088

**Published:** 2026-02-01

**Authors:** Thomas Samson, Maral Aali, Darien McBride, Thomas Arnason, Sharon E. Clarke, Ravi Ramjeesingh, Lisette Gonzalez-Chavez, Yara Azizieh, Mark J. Walsh, Scott M. Livingstone, Stephanie E. Hiebert, Jeanette E. Boudreau, Boris L. Gala-Lopez

**Affiliations:** 1 Faculty of Medicine, Dalhousie University, Halifax, NS B3H 4R2, Canada; th449890@dal.ca (T.S.);; 2 Department of Surgery, Dalhousie University, Halifax, NS B3H 4R2, Canada; 3 Department of Pathology, Dalhousie University, Halifax, NS B3H 4R2, Canada; 4 Department of Radiology, Dalhousie University, Halifax, NS B3H 4R2, Canada; 5 Department of Medicine, Dalhousie University, Halifax, NS B3H 4R2, Canada; 6 Beatrice Hunter Cancer Research Institute, Halifax, NS B3H 0A2, Canada; 7 Department of Microbiology & Immunology, Dalhousie University, Halifax, NS B3H 4R2, Canada

**Keywords:** pancreatic ductal adenocarcinoma, sAXL, AXL receptor tyrosine kinase, CA19-9, prognostic biomarker, preoperative risk stratification, early mortality

## Abstract

Surgical resection combined with chemotherapy offers the best chance of survival in pancreatic ductal adenocarcinoma (PDAC), but many will experience recurrence and early mortality. We examined soluble AXL (sAXL), a blood protein, for its ability to predict 6-month mortality after resection and compared it to CA19-9. Fifty-four patients with PDAC who underwent tumour resection were analyzed to assess biomarker performance and identify optimal cut-off levels. The cut-off for sAXL was 40.26 ng/mL (sensitivity 0.729; specificity 0.643), while it 253.3 U/mL for CA19-9 (sensitivity 0.591; specificity 0.621). Patients with sAXL > 40.26 ng/mL had a non-significant trend toward worse survival (log-rank *p* = 0.088). Univariate Cox regression revealed that high tumour grade (3 + 4) and positive resection margin significantly predicted early mortality. Multivariate Cox regression showed that sAXL > 40.26 ng/mL remained associated with 6-month mortality (hazard ratio 2.42, bootstrap 95% CI 1.15–5.65, *p* = 0.020), independent of high tumour grade (hazard ratio 4.02, bootstrap 95% CI 1.68–13.2, *p* = 0.002). These findings suggest that a preoperative blood test (sAXL) has utility for predicting futile surgery beyond the current standard, CA19-9, and can be incorporated into larger models to assist in risk stratification and follow-up planning.

## Simple Summary

Pancreatic ductal adenocarcinoma (PDAC) is the third leading cause of cancer mortality in Canada. Radical surgery combined with chemotherapy is the only hope for a cure, but many of those undergoing surgery will still recur and die within 6–24 months. We examined soluble AXL (sAXL), a protein shed into the blood from PDAC tumours, for its ability to distinguish risk for death within the first six months post-surgery. Six-month mortality was 2 to 3 times more likely if they had high levels of sAXL in their blood before surgery. This study supports further research into more complex models to predict early mortality after surgery, to guide pre-operative conversations and identify those who should undergo more intensive follow-up after resection or those who would not benefit at all from surgery.

## 1. Introduction

Pancreatic ductal adenocarcinoma (PDAC) is one of the deadliest cancers. It is currently the third leading cause of cancer-related mortality in Canada and is expected to rise further [1,2]. Despite advances in treatment, 5-year survival remains below 10%, mainly due to late diagnosis and aggressive tumour biology [3,4,5,6,7,8,9]. Surgical resection with systemic chemotherapy offers the best chance for long-term survival, but recurrence rates are high, and outcomes remain poor [5,6,7,8]. Clinicians often use the tumour pathological characteristics to predict the worst outcome and to adjust therapeutic strategies. However, there is a lack of consistent data to identify preoperatively which patients are likely to experience an early recurrence despite the best possible treatment.

Currently, CA19-9 is the only FDA-approved blood biomarker; however, it has limited sensitivity and specificity, restricting its use primarily to monitoring rather than early detection or prognostication [10,11]. Moreover, CA19-9 expression depends on the Lewis blood group system, and about 5–10% of patients with PDAC do not express this protein. Numerous investigational biomarkers, including circulating tumour DNA, circulating tumour cells, exosomes, and specific microRNAs, have shown promise for earlier PDAC detection and risk stratification. Still, none are yet in widespread clinical use [12,13,14]. One emerging candidate is AXL, a receptor tyrosine kinase frequently overexpressed in PDAC, that has been correlated with aggressive tumour features and poor prognosis [15,16,17]. Its soluble form (sAXL), released into plasma, has shown promise as a non-invasive biomarker with the potential to improve detection, especially when combined with CA19-9 [11,18,19].

PDAC is also known for presenting a thick desmoplastic stromal matrix, which seems to play an essential role in cancer progression, metastasis, drug resistance and immunosuppression. This stroma is highly heterogeneous and consists of immune cells, fibroblasts, microvasculature, nerves, and an acellular extracellular matrix [20]. Recently, Koay and collaborators described a new quantitative method to characterize the biophysical profiling of PDAC and predict its behaviour [21]. In Koay’s study, the authors defined the interface between the PDAC tumour and the surrounding pancreas parenchyma as seen on regular CT scans. They measured tissue density in Hounsfield Units (HU). The mean HU value within each contour was compared, yielding a difference known as the “delta”. Tumours with high delta had more aggressive biological features, were more likely to develop early distant metastasis, and had shorter overall survival [22,23].

The current study aims to evaluate the performance of the sAXL biomarker and the biophysical profile of PDAC, and to explore its potential use as a pre-surgical predictor of early (6-month) mortality from recurrence in resected PDAC. The current paper is exploratory and serves as a validation step toward the development of robust pre-surgical risk stratification tools.

## 2. Materials and Methods

### 2.1. Study Design

We conducted a retrospective cohort study using prospectively collected biospecimens and clinical data from patients undergoing surgical resection for PDAC. The study was based at a single academic tertiary-care center as part of the Atlantic Cancer Consortium Biobank. All patients had curative-intent pancreatic resection for PDAC, with biospecimen collection and long-term follow-up. No experimental interventions or treatment decisions were included in this study; instead, we performed post hoc biomarker analyses on banked samples and related them to clinical outcomes.

Primary analyses evaluated the associations between plasma biomarker sAXL and CA19-9 levels and patient outcomes. The most established marker, tumour grade, was used as a comparator. We also conducted a subanalysis to establish the biophysical subtype of PDAC and further investigated the associations between the observed deltaHU and patient outcomes.

### 2.2. Study Population

Eligible participants were aged 18 or older and diagnosed with PDAC who underwent surgical resection at the QEII Health Sciences Centre (Halifax, NS, Canada) between January 2018 and December 2023 (REB protocol #1026628). Both patients undergoing upfront surgery and those who received neoadjuvant therapy were included. Patients with other pancreatic neoplasms (such as neuroendocrine tumours, duodenal carcinoma, ampullary adenocarcinoma, cholangiocarcinoma or metastatic lesions to the pancreas) were excluded.

**Figure 1:**
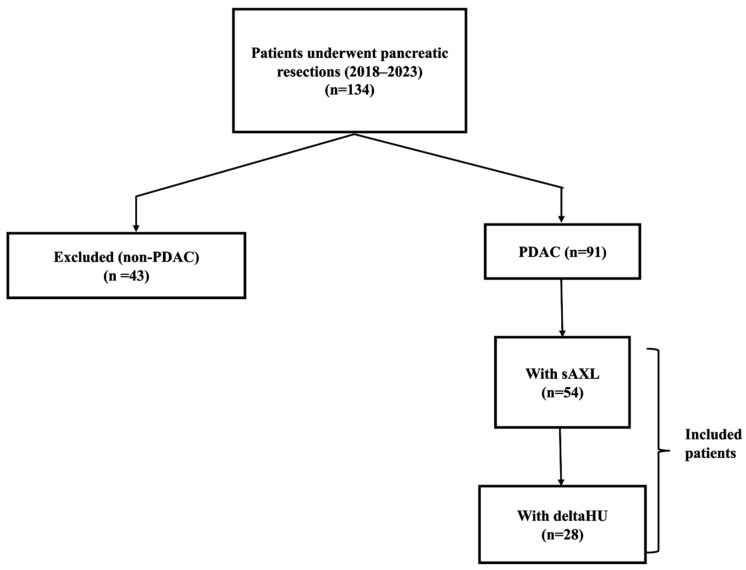
Flow chart for patient selection. PDAC, pancreatic ductal adenocarcinoma; sAXL, soluble AXL; deltaHU, delta Hounsfield Unit.

The cohort initially included 91 PDAC cases that were available in the biobank at the study’s onset. However, this study was only conducted in a limited number of patients due to its exploratory nature. As a result, sAXL was quantified in plasma samples from 54 participants, and the biophysical profile was completed in 28 patients, with an estimated 80% power to detect meaningful differences between subgroups (α = 0.05) (Figure 1). This target sample size was based on preliminary power calculations and the observed effect sizes in prior studies of sAXL and deltaHU in PDAC, ensuring the study was adequately powered for the primary endpoints in the setting of an exploratory pilot study.

### 2.3. Clinical Data Collection

De-identified clinical data were collected and managed using REDCap (Vanderbilt University, Nashville, TN, USA). The following information was captured for each patient at baseline and during follow-up: age, sex, relevant comorbidities, serum CA19-9 level at diagnosis, clinical TNM staging, details of neoadjuvant treatment (if any, including chemotherapy type and duration, or chemoradiation), type of surgical resection performed, and intraoperative findings. Pathology reports were reviewed to extract tumour characteristics: histopathological subtype (confirming PDAC), differentiation, presence of perineural or vascular invasion, margin status, number of lymph nodes examined, number of positive nodes, and a semiquantitative assessment of tumour stromal density.

Clinical outcome data were obtained from follow-up records up to at least 3 years post-surgery for all surviving patients (with 5-year follow-up available for earlier cases). The primary outcome measures were progression-free survival (PFS) and overall survival (OS) at 1, 3, and 5 years postoperatively. PFS was defined as the time from surgery to the first documented tumour recurrence or progression (or death, if it occurred before recurrence). Overall survival was defined as the time from surgery to death from any cause. Patients without an event were censored at the last follow-up. Recurrence (local or distant) was confirmed through imaging and/or biopsy and recorded with the date of diagnosis. In-hospital mortality (within 30 days) was excluded from the analysis.

### 2.4. Tumour Biophysical Subtype

**Figure 2:**
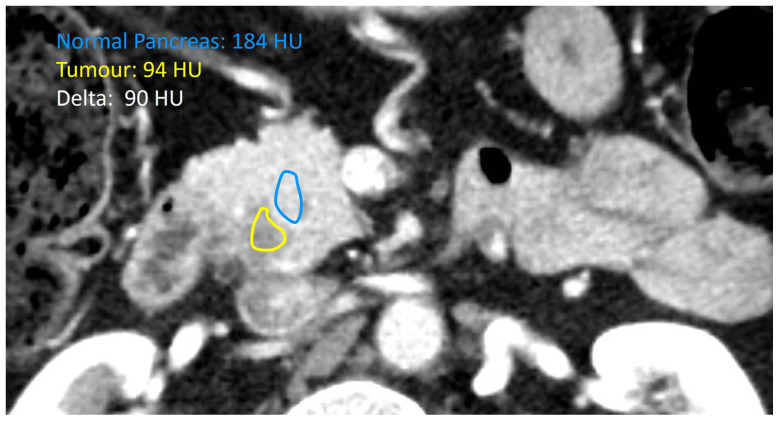
Representative CT slice for one of our biophysical subtype analyses of pancreatic ductal adenocarcinoma showing the interface between tumour and normal pancreatic parenchyma, with the resulting deltaHU (Hounsfield Unit).

Preoperative multiphase CT (and MRI when available) was independently reviewed by a fellowship-trained abdominal radiologist, blinded to the outcomes. The attenuation difference between tumour parenchyma and surrounding stroma (deltaHU) was calculated on preoperative cross-sectional scans and categorized as either low (<26.5 HU) or high (>26.5 HU) based on ROC-derived Youden cutoffs. Figure 2 shows a representative slice from one of the study participants with the assigned deltaHU.

### 2.5. sAXL Quantification

Banked blood specimens were retrieved from the biobank for laboratory analysis. All investigators performing biomarker assays were blinded to the clinical data and outcomes for objectivity. Plasma samples obtained at the time of surgery were analyzed for soluble AXL (sAXL) concentration using a solid-phase Enzyme-Linked Immunosorbent Assay (ELISA). We used a commercially available human AXL ELISA kit (Invitrogen, Carlsbad, CA, USA) according to the manufacturer’s protocol. The absorbance was read at 450 nm. Biomarkers CA19-9 and sAXL were dichotomized into “High and Low” based on ROC-derived Youden cutoffs: 40.26 ng/mL for sAXL and 253.3 (U/mL) for CA19-9.

### 2.6. Statistical Analysis

Statistical analyses were performed using R version 4.3.1 and SPSS version 27. In using R, the following three packages were utilized: “timeROC”, “survival”, and “survminer”. For descriptive statistics, continuous variables were displayed as median [IQR], and categorical variables as as number and percentage (%). Six-month mortality following surgical resection was the primary outcome variable. Time-dependent ROC analyses were performed on sAXL and CA19-9. The AUC was reported with a 95% confidence level, and the Youden index was used to determine the optimal threshold for predicting 6-month mortality. Long-term survival analysis was performed using Kaplan–Meier curves and the log-rank test.

Cox proportional hazards regression was used to estimate hazard ratios for both univariate and multivariable models, which included sAXL and tumour grade. Soluble AXL and grade were selected a priori based on the well-established prognostic strength of cancer grade, as well as the hope of determining whether sAXL was a surrogate for tumour biology explained by grade or had prognostic potential independently. Following visual inspection of the survival plots, the proportional hazards assumption was not violated. This model must be considered exploratory due to the low number of events.

## 3. Results

A total of 134 patients undergoing pancreatic resections during the study period were included in our institutional biobank. Only 91 (70%) were diagnosed as PDAC and were used as our study population. The remaining cases were peri-ampullary carcinomas (6.2%), cholangiocarcinoma (2.3%) and other neoplasms (21.5%).

After sAXL quantification, cases were divided into two groups (high and low) based on the calculated threshold of 40.26 ng/mL. The baseline demographic, surgical, and tumour characteristics are summarized in Table 1 and Table 2. Neoadjuvant chemotherapy was used in 46% of patients, and the most frequent procedure was pancreaticoduodenectomy (76%). Tumour characteristics were similar between groups, including size (*p* = 0.999), differentiation (*p* = 0.787) and degree of local invasion (*p* = 0.741). Recurrence occurred in 28% of patients (*p* = 0.765). Early mortality (≤6 months) occurred in 7 patients (13%), and 12-month mortality occurred in 16 patients (30%).

Time-dependent ROC analyses were performed on both biomarkers, sAXL and CA19-9, to compare their ability to discriminate mortality at 6, 12, and 30 months (Table 3). sAXL outperformed CA19-9 at all time points, and the discriminative power of both biomarkers decreased with increasing time from resection.

**Figure 3:**
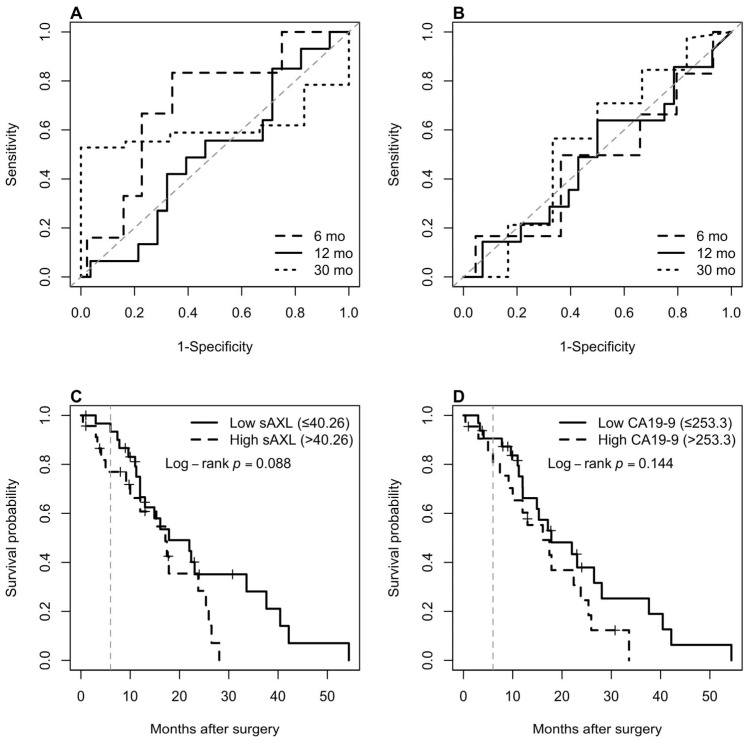
Time-dependent ROC curves for soluble AXL (sAXL) (**A**) and CA19-9 (**B**) at 6-, 12-, and 30-month endpoints. Kaplan–Meier survival curves showing a non-significant trend toward shorter postoperative survival among patients with elevated sAXL (*p* = 0.088) (**C**) and a similar trend for CA19-9 (*p* = 0.144) (**D**). The grey dashed lines indicate the 6-month mark.

Youden indices were calculated to identify an actionable cut-off for each biomarker that balances sensitivity and specificity in discriminating 6-month mortality. The derived cut-offs were 40.26 ng/mL for sAXL, with a sensitivity of 0.73 and specificity of 0.64 (Figure 3A); and 253.3 U/mL for CA19-9, with a sensitivity of 0.59 and specificity of 0.62 (Figure 3B). These values were used in subsequent Cox regression analyses.

When comparing survival between the two groups using the calculated sAXL cutoff, we observed differences in survival trajectories, with a survival advantage for the low sAXL group. However, the difference did not reach statistical significance (log-rank *p* = 0.088; Figure 3C). A similar analysis using the calculated preoperative Ca19-9 cutoff failed to show a significant difference (log-rank *p* = 0.144; Figure 3D).

**Figure 4:**
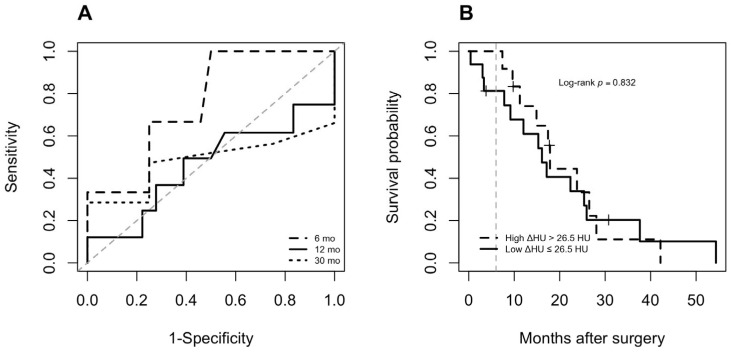
(**A**) Time-dependent ROC curves for delta Hounsfield Unit (deltaHU) based on the calculated 26.5 HU cutoff to predict 6-month postoperative mortality. (**B**) Kaplan–Meier survival curves showing similar postoperative survival between patients with low and high deltaHU (*p* = 0.832). The grey dashed lines indicate the 6-month mark.

We performed a subanalysis of 28 patients with PDAC to estimate the biophysical subtype. Table 2 presents the characteristics of these subjects, divided into high- and low-deltaHU groups based on the calculated 26.5 HU cutoff to predict 6-month postoperative mortality (Figure 4A). However, there were no significant differences in survival when using this threshold (log-rank *p* = 0.832, Figure 4B). There was no association between deltaHU and sAXL.

The identified predictors of 6-month mortality were further analyzed using univariate Cox regression, as shown in Table 4. Only the tumour grade and resection margins were observed to predict early mortality.

A Multivariable Cox regression model, including sAXL >40.26 ng/mL and high-grade (3 + 4), was built to predict 6-month mortality; the results are shown in Table 5.

To further characterize the effects of sAXL independent of tumour grade, sAXL was stratified by tumour grade. Median sAXL levels were 39.0 ng/mL (Q1–Q3: 28.4–55.3) in low-grade tumours (grades 1–2) and 32.4 ng/mL (Q1–Q3: 24.2–41.0) in high-grade tumours (grades 3–4). sAXL also showed no significant difference between grade (high vs. low) when using the Wilcoxon rank-sum test (*p* = 0.38).

## 4. Discussion

Pancreatic cancer remains one of the most lethal malignancies. Despite the best care, up to 45% of resectable patients will die within a year of radical surgery [24], and there is still no accurate method to predict early recurrence among patients. This exploratory study demonstrates the value of the recently described soluble AXL biomarker in identifying subjects at risk of early recurrence and mortality when combined with other widely available tumour indicators, especially pathological grading. As demonstrated by Martinez-Bosch and colleagues, sAXL appears to perform better than the FDA-approved serum CA19-9 for both short- and long-term survival [25].

Previous studies have published ample data supporting the value of several biomarkers in pancreatic cancer, including other carbohydrate antigens such as CA50, CA242, and CA125, which perform well for diagnostic purposes. However, as with CA19-9, they are prone to false positives in patients with cholestatic patterns and have not shown any meaningful predictive value [26]. Other systemic inflammation markers have been shown to correlate with shorter overall survival, including C-reactive protein, albumin, the neutrophil-to-lymphocyte ratio, and the modified Glasgow Prognostic Score (combining albumin and CRP). Yet, they have failed to demonstrate a clear prognostic value [27].

The measurement of KRAS mutations in circulating tumour DNA is also gaining interest as a prognostic tool, particularly in response to chemotherapy. It appears to outperform CA19-9 in identifying patients with poor outcomes [28]. The reality is that many of these new markers are not consistently used in real-world clinical decision-making due to their complexity and cost. Since we have encountered the same limitations in our practice, we were fascinated by sAXL due to its technical simplicity compared to many other biomarkers and its recently published ability to discriminate from other benign pancreatic disorders. Additionally, approximately 10% of the population is unable to synthesize CA19-9 [29,30].

In our study, sAXL demonstrated a stronger ability to prognosticate overall survival than CA19-9, but this property decreased over time from the time of resection. Even though the overall survival difference between the groups with high and low sAXL did not reach statistical significance, there was a trend toward worse survival in patients with an sAXL greater than 40.26 ng/mL (*p* = 0.088, hazard ratio 2.42, 95% CI 1.15–5.65, Wald *p* = 0.020), suggesting that larger studies are warranted to confirm this effect.

Consistent with many other published studies, we observed that high tumour grade (3 + 4) and positive margin were the strongest predictors of 6-month mortality [30,31,32]. Yet, when high grade (3 + 4) and high sAXL were included in a futility risk multivariate model, they both remained significant independent risk factors, suggesting sAXL uncovers unique aggressive tumour biology and is not simply a surrogate for high grade class.

To further study the influence of the tumour microenvironment on early oncological outcomes preoperatively, we performed a sub-analysis within this PDAC cohort of patients whose tumour stromal characteristics were estimated using deltaHU, as previously reported. Subsequently, we evaluated its influence on 6-month mortality [22,23]. The deltaHU failed to demonstrate any association with tumour grading, local invasion, preoperative CA19-9, or sAXL, and did not influence short- or long-term mortality; therefore, it is not helpful in our patient cohort to predict early relapse after surgery.

Our results must be interpreted while accounting for several limitations. This work used a prospective biobank, but the study was conducted retrospectively, using already-collected samples and data, which included only preoperative blood samples. Given the exploratory nature of the study, we included only one center with the hope of detecting meaningful results that would allow launching a larger multicenter project. As such, the sample size is reduced, there is currently no external validation, there are few events per variable, and the confidence intervals are wide. Other limitations include inconsistent preoperative cross-sectional imaging used to calculate the deltaHU, particularly regarding the CT or MRI enhancement protocol, slice width, and overall study quality, which at times prevented completion of biophysical profiling.

## 5. Conclusions

In summary, this study explored the value of preoperative sAXL in predicting early recurrence after surgical resection of PDAC. We observed that an sAXL level greater than 40.26 ng/mL was predictive of early recurrence and poor overall survival, especially when combined with tumour grade and resection margins. These findings, although exploratory, show promise for future work on sAXL’s utility as a pre-surgical blood test that could be integrated with other clinical and imaging variables to identify high-risk surgical candidates, enabling more informed preoperative conversations and enhanced follow-up.

Further studies with a larger sample size are strongly recommended, as they may improve the standalone predictive value of sAXL, even without tumour characteristics from pathology analysis. Likewise, incorporating such predictors may further contribute to the design of more personalized treatments, optimizing treatment based on expected survival, and providing objective information to predict a futile invasive procedure.

## Figures and Tables

**Table 1:** Baseline clinicopathologic characteristics according to pre-operative soluble AXL (sAXL) group (≤40.26 ng/mL vs. >40.26 ng/mL).

Characteristic	Low sAXL ≤ 40.26	High sAXL > 40.26	*p*-Value
Sex			0.593
Male	16 (52%)	10 (43%)	
Female	15 (48%)	13 (57%)	
Age (year)			0.265
Median (Q1, Q3)	65 (59, 69)	79 (60, 76)	
CA19-9 (U/mL)			0.800
Median (Q1, Q3)	157 (66, 714)	216 (48, 816)	
Neoadjuvant type			0.324
CTX	16 (52%)	8 (35%)	
RTX	0 (0%)	0 (0%)	
Combined	1 (3%)	0 (0%)	
Neither	15 (45%)	15 (65%)	
Surgery type			>0.999
PD	23 (74%)	18 (78%)	
Distal pancreatectomy	2 (6%)	1 (4%)	
Total pancreatectomy	6 (19%)	4 (17%)	
Tumour size			0.822
<2 cm	5 (16%)	2 (13%)	
2–4 cm	20 (65%)	16 (70%)	
>4 cm	6 (19%)	4 (17%)	
Tumour differentiation			0.787
Well	5 (16%)	4 (22%)	
Moderate	20 (65%)	15 (65%)	
Poor	6 (19%)	3 (13%)	
Undifferentiated	0 (0%)	0 (0%)	
Lymph-node-involvement			0.741
N0	4 (13%)	4 (17%)	
N1 (1–3)	13 (42%)	12 (52%)	
N2 (≥4)	13 (42%)	7 (30%)	
Lymph-vascular-invasion			0.863
No	5 (16%)	5 (22%)	
Yes	25 (81%)	17 (74%)	
Perineural invasion			0.205
No	5 (16%)	1 (4%)	
Yes	26 (84%)	21 (91%)	
Resection margins			0.867
Negative	22 (71%)	16 (70%)	
Positive	8 (26%)	7 (30%)	
Adjuvant CTX	20 (67%)	14 (61%)	0.775
sAXL (ng/mL)			**<0.001**
Median (Q1, Q3)	28 (23, 34)	56 (45, 62)	
Recurrence	9 (31%)	6 (26%)	0.765
Death ≤ 6 months	2 (6%)	5 (22%)	0.122
Death ≤ 12 months	9 (29%)	7 (30%)	>0.999

Notes: Values are presented as n (%) or median (Q1, Q3). Statistical comparisons were performed using Fisher’s exact or Wilcoxon rank-sum tests as appropriate. Percentages may not sum to 100% due to missing or unreported data for chemotherapy (CTX), radiotherapy (RTX), and pancreaticoduodenectomy (PD). Bolded *p*-values indicate significant differences.

**Table 2:** Baseline clinicopathologic characteristics according to delta Hounsfield Unit (ΔHU) group (≤26.5 HU vs. >26.5 HU).

Characteristic	Low ΔHU ≤ 26.5	High ΔHU > 26.5	*p*-Value
Sex			>0.999
Male	5 (42%)	7 (44%)	
Female	7 (58%)	9 (56%)	
Age (y)			0.767
Median (Q1, Q3)	67 (61, 71)	65 (59, 75)	
CA19-9 (U/mL)			0.834
Median (Q1, Q3)	190 (109, 395)	234 (42, 1 145)	
Neoadjuvant type			0.687
CTX	3 (25%)	6 (38%)	
RTX	0 (0%)	0 (0%)	
Combined	0 (0%)	0 (0%)	
Neither	9 (75%)	10 (62%)	
Surgery type			0.101
PD	6 (50%)	13 (81%)	
Distal pancreatectomy	2 (17%)	0 (0%)	
Total pancreatectomy	4 (33%)	3 (19%)	
Tumour size			0.714
<2 cm	2 (17%)	1 (6%)	
2–4 cm	8 (67%)	11 (69%)	
>4 cm	2 (17%)	4 (25%)	
Tumour differentiation			0.088
Well	1 (8%)	4 (25%)	
Moderate	11 (92%)	8 (50%)	
Poor	0 (0%)	4 (25%)	
Undiff	0 (0%)	0 (0%)	
Lymph node involvement			0.542
N0	4 (33%)	2 (12%)	
N1 (1–3)	4 (33%)	6 (38%)	
N2 (≥4)	4 (33%)	8 (50%)	
Lymph-vascular invasion			>0.999
No	2 (17%)	2 (12%)	
Yes	10 (83%)	14 (88%)	
Perineural invasion			>0.999
No	1 (8%)	1 (6%)	
Yes	11 (92%)	15 (94%)	
Resection margins			>0.999
Negative	8 (67%)	10 (62%)	
Positive	4 (33%)	6 (38%)	
Adjuvant CTX	7 (58%)	12 (75%)	0.432
ΔHU (HU)			**<0.001**
Median (Q1, Q3)	18 (−2, 23)	40 (32, 48)	
Recurrence	4 (36%)	5 (31%)	>0.999
Death ≤ 6 months	0 (0%)	3 (19%)	0.238
Death ≤ 12 months	3 (25%)	5 (31%)	>0.999

Notes: Values are presented as n (%) or median (Q1, Q3). Statistical comparisons were performed using Fisher’s exact or Wilcoxon rank-sum tests as appropriate. Percentages may not sum to 100% due to missing or unreported data for chemotherapy (CTX), radiotherapy (RTX), and pancreaticoduodenectomy (PD). Bolded *p*-values indicate significant differences.

**Table 3:** Time-dependent ROC analysis of soluble AXL (sAXL) and CA19-9 for discerning early mortality (6, 12, 30 months) with 95% confidence intervals (n = 54).

Timepoint	Biomarker	AUC	95% CI	Deaths at Timepoint (n)
6 months	sAXL	0.711	0.508–0.914 *	**7**
6 months	CA19-9	0.472	0.214–0.729	7
12 months	sAXL	0.500	0.319–0.681	16
12 months	CA19-9	0.488	0.300–0.676	16
30 months	sAXL	0.610	0.443–0.778	33
30 months	CA19-9	0.553	0.262–0.844	33

Notes: Abbreviations: AUC, area under the receiver operating characteristic curve; CI, confidence interval; PDAC, pancreatic ductal adenocarcinoma; (*) performance above chance.

**Table 4:** Univariate Cox Regression Analyses Predicting 6-Month Mortality post pancreatic ductal adenocarcinoma (PDAC) surgical resection with 95% confidence intervals.

Predictor (Comparator)	HR	95% CI	*p*-Value
sAXL (per ng/mL increase)	1.01	0.99–1.02	0.48
High sAXL (>40.26 ng/mL vs. ≤40.26)	1.61	0.87–2.95	0.127
CA19-9 (per U/mL increase)	1.00	1.00–1.00	0.40
High CA19-9 (>253.3 U/mL vs. ≤253.3)	1.47	0.81–2.67	0.21
DeltaHU (per unit increase)	1.89	0.86–4.20	0.12
High DeltaHU (≥40 vs. <40)	0.53	0.24–1.17	0.12
High grade (3–4 vs. 1–2)	1.95	1.03–3.71	0.041 *
Margin positive (vs. negative)	2.16	1.15–4.05	0.017 *
LN N1 (vs. N0)	0.61	0.33–1.41	0.12
LN N2 (vs. N0)	1.36	0.76–2.45	0.304
Tumour size 2–4 cm (vs. <2 cm)	0.87	0.47–1.62	0.66
Tumour size >4 cm (vs. <2 cm)	1.18	0.59–2.37	0.64
Tumour site: tail (vs. head/neck)	0.56	0.17–1.87	0.35
Tumour site: body (vs. head/neck)	2.26	0.53–9.69	0.27
LVI present (vs. absent)	1.06	0.48–2.30	0.89
PNI present (vs. absent)	1.14	0.40–3.26	0.81
Age (per year increase)	0.98	0.95–1.01	0.21
Female sex (vs. male)	0.89	0.51–1.57	0.69
Adjuvant therapy (yes vs. no)	0.82	0.46–1.44	0.49
Surgery: distal (vs. Whipple)	0.54	0.12–2.34	0.41
Surgery: total (vs. Whipple)	0.83	0.41–1.68	0.61
sAXL (per ng/mL increase)	1.01	0.99–1.02	0.48

Notes: HR = hazard ratio; CI = confidence interval. Reference categories are specified in parentheses. Variables with too few events were not estimable and excluded. *p* < 0.05 is considered statistically significant (*).

**Table 5:** Multivariable Cox Regression model including (1) soluble AXL (sAXL) > 40.26 ng/mL and (2) High grades 3 + 4, predicting 6-Month mortality following pancreatic ductal adenocarcinoma (PDAC) surgical resection.

Predictor	HR	95% CI (Bootstrap, 2000 Reps)	Wald *p*-Value
High sAXL (>40.26 ng/mL)	2.42	1.16–5.65	0.020 *
High grade (3–4 vs. 1–2)	4.02	1.68–13.2	0.002 *

Notes: HR = hazard ratio; CI = confidence interval. Wald *p*-values are reported; 95% CI derived from 2000 bootstrap replications for robustness. * indicates a significant difference.

## Data Availability

The data presented in this study are available upon request from the corresponding author due to ethical reasons.

## Abbreviations

The following abbreviations are used in this manuscript:

AUC	Area under the curve
CI	Confidence interval
HR	Hazard ratio
KM	Kaplan–Meier
OR	Odds ratio
PDAC	Pancreatic ductal adenocarcinoma
ROC	Receiver operating characteristic
sAXL	Soluble AXL
